# Effect of pine bark extract and its phenolic compounds on selected pathogenic and probiotic bacterial strains

**DOI:** 10.3389/fnut.2024.1381125

**Published:** 2024-03-27

**Authors:** Teresa Sánchez-Moya, Rubén López-Nicolás, Patricia Peso-Echarri, Carlos A. González-Bermúdez, Carmen Frontela-Saseta

**Affiliations:** Department of Food Science and Nutrition, Faculty of Veterinary Sciences, Regional Campus of International Excellence Campus Mare Nostrum, University of Murcia, Murcia, Spain

**Keywords:** pine bark extract, phenolic compounds, antimicrobial activity, pathogenic bacteria, probiotics, inflammatory bowel disease

## Abstract

**Introduction:**

Inflammatory bowel disease (IBD) comprises a heterogeneous group of chronic diseases as ulcerative colitis (UC) and Crohn’s disease (CD). IBD is the result of a dysregulation of intestinal homeostasis with a host’s loss of tolerance toward normal enteric microflora. Plant-based extracts as phenolic compounds can play a role by modulating the intestinal inflammation response.

**Methods:**

The *in vitro* antimicrobial activity of French maritime pine bark extract (PBE) and its phenolic constituents has been investigated in this study. Furthermore, the ability of PBE and phenolic compounds (caffeic acid, chlorogenic acid, ferulic acid, gallic acid and taxifolin) to modulate the microbiota has been assessed.

**Results:**

Phenolic compounds and PBE showed a great inhibitory effect on the pathogens growth at the highest concentration assessed (1.25 mg/mL). The growth of *E. sakazakii* and *E. faecalis* were affected by the effect of caffeic acid and ferulic acid. Taxifolin showed a very strong activity against *Listeria* sp. (with a reduction ~98%). Gallic acid revealed antibacterial effect on *S. aureus* at different concentrations. The inhibitory effect of PBE was highly significant on the growth of *E. coli* O157:H7. PBE, caffeic acid and chlorogenic acid seem to provide the greatest beneficial effect on the probiotic bacteria. However, the highest concentrations of taxifolin may have impaired the growth of beneficial microbiota.

**Conclusion:**

Present findings could be of interest for considering PBE and/or its phenolic constituents as protectors against gastrointestinal disturbances which lead to ulcerative colitis and Crohn’s disease.

## Graphical abstract

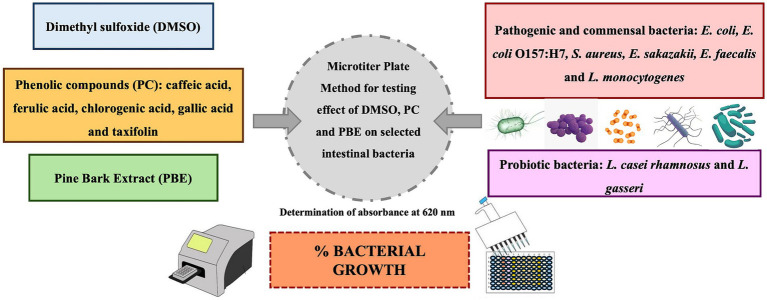

## 1 Introduction

Inflammatory bowel disease (IBD) is a group of chronic inflammatory conditions that affects gastrointestinal tract and can be associated to a dysregulation of immune system and microbiota (1–4). IDB is a heterogeneous complex state of chronic intestinal inflammation characterized by interactions among gut microbiota, host genetic and environmental factors that could influence immune system (1, 3, 4). IBD is clinically divided into two subtypes: ulcerative colitis (UC) and Crohn’s disease (CD) depending on symptoms, disease location and histopathological features (2, 4, 5). Common symptoms includes abdominal pain, diarrhea and melena, abdominal cramps, fever, fatigue, anemia, weight loss and hematochezia (2, 6). One of the worst complications of IBD is the development of colorectal cancer since a chronic inflammation could be the trigger of neoplastic progression in multiple areas of the colon (7).

Inflammation is a type of nonspecific immune response that defends the body against the constant threat of a myriad of organism and chemical substances from the surrounding environment. Because of this permanent antigenic pressure, intestinal mucosa is adapted to work under intense, yet “physiological,” conditions relying on tight cellular and molecular control mechanism (8). IBD is characterized by reductions in epithelial integrity and increases of mucosal permeability which lead to an unbalanced production of cytokine/chemokine in favor of proinflammatory cytokines (9). Current evidence indicates that chronic intestinal inflammation could be due to the inability of the immune system to regulate gut microbiota, as well as alterations of influx of inflammatory cells via chemokines (9). In some individuals, this delicate balanced state is altered, becomes excessive, which triggers the pathogenesis of these chronic inflammatory disorders.

Consumption of ultra-processed foods, a hallmark of the Western diet, creates an enhanced environment for the selection of microorganisms that promote diet-related diseases through diet-microbiome-host interactions (10). In this sense, gut dysbiosis can be defined as an imbalance in gut microbiota characterized by a reduction in microbial diversity and increases of proinflammatory species. Dysbiosis is related to the development of some pathologies such as obesity, atherosclerosis, type-2 diabetes, and IBD. Loss in microbiota diversity, a reduction of beneficial bacteria and/or increases of pathogenic bacteria may be observed in IBD patients (3). Even though IBD is caused by an altered relationship between gut microbiota and host immune system, the specific mechanism underlying intestinal microbiota disorder is not clear. Possibly, gut microbiota dysbiosis might lead to a disruption in immune tolerance and hence may induce IBD (6). An altered microbiota is associated with increases of the intestinal permeability and the translocation of harmful bacterial components such as lipopolysaccharide (LPS) and toxins, which can lead to a systemic inflammation (11). LPS may leave the intestinal environment, migrating toward the bloodstream to activate cells of the immune system (macrophages, neutrophils, and dendritic cells) through recognition of the Toll-like receptor (TLR). Eventually, proinflammatory cytokines such as IL-1α, IL-1β, IL-6 and TNF-α would be produced, leading to metabolic endotoxemia (12).

In recent years, especially in Western societies, there is a great demand for nutritional supplements of natural origin due to their alleged health benefits. The consumption of plant-based supplements as phytochemicals can be useful for the prevention of several pathologies (13). Plant polyphenols, with more than 8,000 identified compounds, constitute one the largest and most ubiquitous groups of secondary metabolites that are a part of human diet (14). Major polyphenols can be classified into five structural groups: phenolic acids, flavonoid, anthocyanins, stilbenes and lignans (15). Evidence suggests that the consumption of polyphenol rich-foods plays a beneficial role in the prevention of cancer, coronary heart disease, obesity and inflammation, among others (16). Furthermore, a growing body of evidence suggests that phenolic compounds could play a role in modulating the intestinal inflammation response (17). Polyphenols not absorbed during digestion could be catabolized by gut microbiota increasing anti-inflammatory metabolites (3). Hence, *in vivo,* and *in vitro* studies have demonstrated the beneficial effect of phenolic compounds in preventing and improving symptoms of IBD. These protective effect are mediated through multiple mechanisms: reducing oxidative stress, modulating gut microbiota diversity, protecting gut barrier and through immune modulation (3). Furthermore, therapeutic manipulation of the intestinal flora offers considerable promises for treating IBD (18). Phenolic compounds have received wide attention, not only for their antioxidant and anticarcinogenic capacities, but their antimicrobial activity. These compounds can interact with gut microbiota, modulating the microbial population through the gastrointestinal tract. Phenolic compounds can stimulate the growth of probiotic bacteria or change the gut microbiota composition in favor of beneficial bacteria as *Lactobacillus* spp., *Bifidobacterium* spp., *Akkermansia muciniphila* and *Faecalibacterium prausnitzii*. Bacteriostatic or bactericidal effect of these compounds depend on the bacterial strain and the polyphenol structure (19). Several studies have demonstrated the prebiotic potential of polyphenols in human and in animals. For instance, tea phenolic compounds and their derivates have significantly reduced the growth of pathogenic bacteria as *Clostridium perfringens, Clostridium difficile* and *Bacteroides* spp. (20). In general, enteropathogenic bacteria such as *Staphylococcus aureus* is very sensible to phenolic compounds, while the probiotic bacteria *Lactobacillus casei rhamnosus* is seems to be less sensitive (21).

Natural products have been used since time immemorial due to their health benefits. Subsequently, they have been used to discover new drugs contributing to the development of pharmaceutical products (22). The bark, pollen, and needles of many species of pine tree have been employed as a useful source of natural products. The first use of pine bark extract (PBE) was described in 16th century by a French explorer who used it for its effects on scurvy. In the past, PBE was considered a waste product of the wood industry, however, now it is considered a rich source of bioactive compounds (23). In this connection, an extract obtained from the bark of the French maritime pine (*Pinus pinaster* Aiton) has been reported as a concentrated source of water-soluble polyphenols, mainly procyanidins, phenolic acids, cinnamic acids and their glycosides, and taxifolin (24). Owing to the basic chemical structure of its components, the most important feature of the PBE is the antioxidant and antimicrobial activity (25–27). Procyanidins contained in the extract are potent quenchers of reactive oxygen species (ROS) (23). A recent research have proved that the pine bark polar extracts showed a strong reducing power and 2,2-diphenyl-1-picrylhydrazyl (DPPH) and 2,2-azinobis-(3-ethyl-benzothiazoline-6-sulfonic acid (ABTS) radical scavenging effects compared to natural antioxidants (28). In addition it can be used as a nutritional supplement due to its anti-inflammatory and immunomodulatory effect (13, 24, 29, 30). PBE could be useful to prevent diseases as atherosclerosis, hypertension, diabetes and cancer (30). The antibacterial activity of PBE has been proved against Gram-negative bacteria (*E. coli, K, pneumoniae, P. aeruginosa, Helicobacter pylori*) and Gram-positive bacteria (*E. faecalis, C. perfringens, S. aureus*) (23, 27). PBE not only influences bacteria but also viruses. Some researchers have highlighted the effect of different pine extracts against infections such as Epstein–Barr virus and human immunodeficiency virus type-1 by different mechanism of action such as inhibiting the transcription of immediate early genes and lytic cycle, and blocking the binding of this virus to human cells, respectively (23).

Therefore, the objective of this study was to determine the *in vitro* effect of PBE and its phenolic compounds on gut microecology by investigating their impact on the growth of bacteria commonly present in the human gastrointestinal tract (pathogens, commensals, and probiotics).

## 2 Materials and methods

### 2.1 Bacterial strains and culture media

Eight common pathogenic, commensal, and probiotic intestinal bacteria were chosen in this study as representative of intestinal microflora in a similar form to López-Nicolás et al. (23). *Escherichia coli* (NUTBRO collection) was selected as commensal intestinal bacteria, and it was grown on nutrient Müeller-Hinton broth at 37° C for 24 h. Aerobic pathogens assessed included *Escherichia coli* O157:H7 (DMSZ 13526), *Staphylococcus aureus* (NUTBRO collection), *Enterobacter sakazakii* (CECCT 858) and *Enterococcus faecalis* (DSMZ 20478) grown on nutrient Müeller-Hinton broth at 37° C for 24 h. Other pathogen, as *Listeria monocytogenes* (NUTBRO collection) was included, and it was grown in brain heart infusion (BHI) broth at 37° C for 24 h. The probiotics *Lactobacillus gasseri* (DSMZ 20077) and *L. casei rhamnosus (*ATCC 6469) were maintained on the Mann Rogosa and Sharpe medium (MRS) under anaerobic conditions at 37° C for 48 h. To prepare the bacterial inoculums 10^8^ CFU (0.5 McFarland scale) were taken in a sterile tube containing 2 mL NaCl. Bacterial inoculums were diluted 1:1000 in peptone water, achieving a final concentration of 10^5^ CFU/mL (31).

### 2.2 Phenolic compounds and DMSO

Phenolic compounds were purchased in Sigma-Aldrich, St. Louis (United States), and stored as stocks of 25 mg/mL in dimethyl sulfoxide (DMSO) at −20° C. Compounds used in this study were: *i.* Hydroxycinnamic acids: caffeic acid, chlorogenic acid, gallic acid and ferulic acid. *ii.* Flavonoids: taxifolin. *Iii.* Pine Bark Extract (Pycnogenol^®^) was purchased in Horphag Research (Geneve, Switzerland). Each phenolic compound, including PBE, was assessed at different concentrations (0.02, 0.04, 0.07, 0.15, 0.3, 0.6 and 1.25 mg/mL). DMSO was assessed at 0, 0.08, 0.16, 0.3, 0.7, 1.25, 2.5 and 5%. DMSO was tested to determine the non-toxic effect on bacteria (31).

### 2.3 Effect of phenolic compounds on intestinal bacterial growth

Antimicrobial activity of phenolic compounds, PBE and DMSO were tested by using 96-well microtiter plate method similarly to López-Nicolás et al. and Chaalal et al. (24). Seven 96-well microtiter plates were prepared for each bacterium at different concentrations of caffeic acid, chlorogenic acid, gallic acid, ferulic acid, taxifolin and PBE (dissolved in different concentrations of DMSO). Microtiter plate method was also used to test DMSO against different bacteria. Growth was compared to a positive control (without chemical compound) to determine the activity of the different chemicals tested. After 16 h of incubation at 37°C, absorbance was measured spectrophotometrically at 620 nm (Spectrophotometer Evolution 300, Thermo Scientific, United States). The antibacterial effect of different compounds was determined as other researches have described previously (31). All tests were performed in a biological triplicate (3 different 96-well plates) and methodological sextuplicate (6 wells per each 96-well plate and concentration).

### 2.4 Statistical analysis

All the data were expressed as mean ± standard deviation (SD) of six replicates. Analysis of variance was performed by ANOVA procedures. Significant differences between means were determined by Tukey’s pairwise comparison test. Values of *p* ≤ 0.05 were considered as statistically significant. All statistical analyses were performed with the Statistical Package for the Social Sciences (version 21.0; SPSS).

## 3 Results and discussion

The growth response (% growth or % inhibition) of eight bacterial strains was measured in presence of different phenolic acids, PBE and DMSO, at increasing concentrations (0–1.25 mg/mL for phenolic compounds and PBE, and 0–5% and DMSO). Selected concentrations were based on previous studies (20, 31, 32). In general, the higher concentration of phenolics compounds, the more inhibition of bacterial growth (33–36). However, differences may be found depending on the phenolic compound and the bacterial strain.

**Figure 1:**
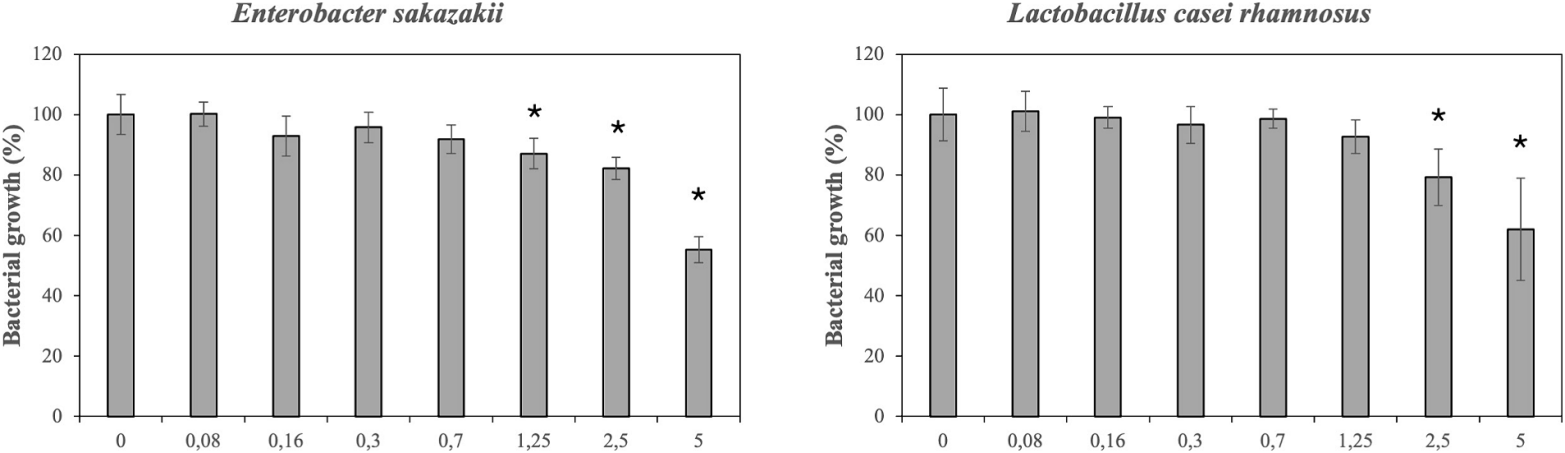
Effect of different concentrations of dimethyl sulfoxide (DMSO) on growth/inhibition of *enterobacter sakazakii* and *Lactobacillus casei rhamnosus*.

Figure 1 illustrates the effect of DMSO (control) on bacterial growth. In general, tested concentrations of DMSO not completely inhibited the bacterial growth. However, the growth of *E. sakazakii* was reduced at concentrations from 1.25 to 5%. Higher concentrations of DMSO (2.5 and 5%) significantly affected the growth of *L. casei rhamnosus.* In addition, *E. coli, S. aureus* and the probiotic *L. gasseri* were significantly affected at the highest concentrations of DMSO (5%; data not shown). The growth of *E. faecalis, E. coli* O157:H7 and *L. monocytogenes* was not significantly affected. Overall, bacterial growth was not greatly compromised by DMSO.

Results after comparing the growth of pathogenic and probiotic bacteria in absence/presence of phenolic compounds are shown in Tables 1–4. Different bacterial strains were incubated with increasing concentrations of phenolic compounds and PBE. Nevertheless, in many cases, the results after incubation with the lowest concentrations (0.02, 0.04 and 0.07) were negligible. In view of this, the results are presented based on concentrations of phenolic compounds from 0.15 to 1.25%.

**Table 1:** Effect of caffeic acid, chlorogenic acid and gallic acid on 16 h growth/inhibition of intestinal microorganism.

	Bacterial strain						
*Phenolic compound*	*mg/mL*	*E. sakazakii*	*E. faecalis*	*E. coli*	*E. coli O157:H7*	*L. monocytogenes*	*S. aureus*
*Caffeic acid*	0	100.00 ± 5.80	100.00 ± 7.71	100.00 ± 4.40	100.00 ± 3.53	100.00 ± 3.38	100.19 ± 2.04
	0.15	78.19 ± 2.49*	89.94 ± 9.15	93.92 ± 2.41	105.22 ± 1.96	92.79 ± 3.68	89.97 ± 5.19*
	0.3	60.69 ± 1.39*	80.42 ± 9.15*	98.28 ± 6.77	101.74 ± 3.21	90.09 ± 7.18*	85.78 ± 5.03*
	0.6	28.00 ± 4.28*	70.97 ± 1.52*	94.45 ± 4.44	91.08 ± 4.18	77.46 ± 1.24*	81.01 ± 5.61*
	1.25	9.85 ± 1.71*	7.78 ± 2.59*	48.71 ± 7.89*	26.07 ± 4.60*	51.86 ± 2.48*	60.07 ± 4.47*
*Chlorogenic acid*	0	100.00 ± 6.92	100.00 ± 4.14	100.00 ± 2.90	100.00 ± 6.48	100.00 ± 14.77	99.36 ± 6.28
	0.15	94.06 ± 7.02	93.98 ± 8.82	83.97 ± 5.96*	102.49 ± 11.36	90.82 ± 7.33	100.20 ± 2.65
	0.3	83.83 ± 4.76*	81.87 ± 3.71*	84.53 ± 7.50*	92.41 ± 8.90	90.65 ± 3.84	92.60 ± 3.31
	0.6	84.13 ± 8.34*	66.71 ± 3.94*	87.90 ± 2.40*	76.40 ± 25.61	75.80 ± 2.71*	79.07 ± 2.21*
	1.25	86.27 ± 4.56*	40.75 ± 3.23*	65.37 ± 0.84*	51.98 ± 16.72*	65.10 ± 10.16*	53.85 ± 3.64*
*Gallic acid*	0	99.77 ± 2.87	102.88 ± 4.69	100.00 ± 2.52	100.00 ± 11.42	102.80 ± 7.45	95.38 ± 20.39
	0.15	88.51 ± 10.99	72.48 ± 4.65*	92.07 ± 5.98	95.52 ± 4.26	100.73 ± 7.94	8.51 ± 1.08*
	0.3	83.04 ± 10.26	76.00 ± 5.10*	33.02 ± 37.15*	98.19 ± 1.54	101.85 ± 3.32	10.38 ± 1.03*
	0.6	51.63 ± 31.47*	73.27 ± 2.91*	13.41 ± 2.3*	99.30 ± 1.69	86.45 ± 3.26*	12.95 ± 3.17*
	1.25	6.78 ± 2.09*	68.18 ± 4.54*	29.98 ± 1.32*	94.43 ± 6.4	68.92 ± 4.86*	7.27 ± 2.82*

Results are shown as mean of percentages ± SD (*n* = 6) compared to bacterial growth in the culture media (31). *For each microorganism, indicates statistical differences (*p* ≤ 0.05) between various concentrations against the positive control (0 mg/mL) within each phenolic compound.

**Table 2:** Effect of ferulic acid and taxifolin on 16 h growth/inhibition of intestinal microorganism.

		Bacterial strain					
*Phenolic compound*	*mg/mL*	*E. sakazakii*	*E. faecalis*	*E. coli*	*E. coli O157:H7*	*L. monocytogenes*	*S. aureus*
*Ferulic acid*	0	99.23 ± 15.35	100.00 ± 13.19	100.00 ± 8.47	100.00 ± 9.45	100.00 ± 12.89	100.77 ± 2.93
	0.15	57.05 ± 4.11*	92.39 ± 4.91	86.14 ± 3.96*	89.14 ± 15.74	98.38 ± 6.50	100.87 ± 2.90
	0.3	44.03 ± 2.37*	79.93 ± 4.65*	69.75 ± 8.03*	89.63 ± 9.14	95.27 ± 3.76	97.47 ± 4.77
	0.6	28.97 ± 6.03*	63.20 ± 3.81*	68.25 ± 7.85*	86.25 ± 5.64	88.87 ± 5.86	88.14 ± 2.03*
	1.25	10.33 ± 2.96*	8.03 ± 3.59*	46.20 ± 7.49*	43.01 ± 6.76*	46.70 ± 5.75*	65.11 ± 4.13*
*Taxifolin*	0	100.00 ± 8.67	100.00 ± 1.91	100.00 ± 4.97	100.00 ± 12.05	98.97 ± 8.20	100.33 ± 2.78
	0.15	87.74 ± 4.59	84.82 ± 5.44*	93.97 ± 5.08	95.72 ± 17.37	99.74 ± 2.81	110.98 ± 2.59*
	0.3	61.08 ± 7.46*	81.45 ± 3.28*	80.42 ± 7.40*	98.86 ± 2.14	94.11 ± 4.75	98.08 ± 2.22
	0.6	57.77 ± 17.98*	64.47 ± 4.47*	46.92 ± 3.67*	86.07 ± 13.86	79.77 ± 8.51*	80.96 ± 1.84*
	1.25	31.01 ± 4.42*	28.67 ± 4.05*	12.45 ± 1.67*	67.77 ± 11.84*	2.81 ± 1.40*	58.84 ± 5.64*

Results are shown as mean of percentages ± SD (*n* = 6) compared to bacterial growth in the culture media (31). *For each microorganism, indicates statistical differences (*p* ≤ 0.05) between various concentrations against the positive control (0 mg/mL) within each phenolic compound.

**Table 3:** Effect of caffeic acid, chlorogenic acid and gallic acid on 16 h growth/inhibition of probiotic bacteria.

		Bacterial strain	
*Phenolic compound*	*mg/mL*	*L. casei rhamnosus*	*L. gasseri*
*Caffeic acid*	0	100.00 ± 9.35	100.00 ± 13.88
	0.15	103.66 ± 8.83	82.58 ± 6.43
	0.3	107.24 ± 7.46	71.93 ± 13.05*
	0.6	104.40 ± 8.60	128.67 ± 6.16*
	1.25	95.41 ± 10.21	110.26 ± 7.29
*Chlorogenic acid*	0	100.00 ± 9.17	100.00 ± 9.73
	0.15	98.47 ± 6.99	102.25 ± 21.38
	0.3	98.58 ± 14.36	91.47 ± 6.82
	0.6	98.58 ± 4.00	113.21 ± 12.93
	1.25	101.59 ± 1.84	101.53 ± 1.89
*Gallic acid*	0	100.54 ± 9.41	100.47 ± 7.68
	0.15	100.27 ± 9.97	96.85 ± 13.25
	0.3	82.07 ± 7.39*	99.03 ± 7.51
	0.6	99.39 ± 10.53	109.09 ± 3.91
	1.25	65.58 ± 7.12*	101.66 ± 2.99

Results are shown as mean of percentages ± SD (*n* = 6) compared to bacterial growth in the culture media (31). *For each microorganism, indicates statistical differences (*p* ≤ 0.05) between various concentrations against the positive control (0 mg/mL) within each phenolic compound.

**Table 4:** Effect of ferulic acid and taxifolin on 16 h growth/inhibition of probiotic bacteria.

	Bacterial strain		
*Phenolic compound*	*mg/mL*	*L. casei rhamnosus*	*L. gasseri*
*Ferulic acid*	0	100.00 ± 8.47	100.00 ± 3.79
	0.15	87.13 ± 10.53	98.38 ± 6.50
	0.3	91.66 ± 7.94	95.27 ± 3.76
	0.6	88.23 ± 14.30	88.87 ± 5.86
	1.25	73.62 ± 8.04*	46.70 ± 5.75*
*Taxifolin*	0	100.00 ± 29.50	98.97 ± 8.20
	0.15	84.90 ± 17.89	99.74 ± 2.81
	0.3	74.45 ± 18.49	94.11 ± 4.75
	0.6	88.43 ± 5.22	79.77 ± 8.51*
	1.25	10.32 ± 0.58*	2.81 ± 1.40*

Results are shown as mean of percentages ± SD (*n* = 6) compared to bacterial growth in the culture media (31). *For each microorganism, indicates statistical differences (*p* ≤ 0.05) between various concentrations against the positive control (0 mg/mL) within each phenolic compound.

Table 1 shows the inhibition of the growth of *E. sakazakii* by caffeic acid (0.15–1.25 mg/mL). The inhibition of bacterial growth at the highest concentration of caffeic acid was fairly considerable (almost 90%) and dose dependent. In addition, the maximum inhibitory effect of caffeic acid was observed in the case of *E. faecalis* at concentrations of 1.25 mg/mL showing reductions of 92.22%, similarly to *E. sakazakii*. Caffeic acid has demonstrated a moderate inhibitory effect against *Listeria* at higher concentrations. Specifically, 1.25 mg/mL of caffeic acid reduced the growth of *L. monocytogenes* almost 50%. The development of the pathogen bacteria *E. coli* O157:H7 was significantly affected at the highest concentrations. However, it is worth highlighting that it was inhibited almost 74%. The probiotic strain *L. gasseri* was only inhibited by caffeic acid at 0.3 mg/mL, meanwhile higher concentrations seemed to enhance its growth. *L. casei rhamnosus* was not affected (Table 3), similarly that results observed by other researches (20, 31).

Chlorogenic acid had the strongest inhibitory effect (near to 60%) on the growth of *E. faecalis* at the highest concentration (1.25 mg/mL). Furthermore, *E. coli* O157:H7, *L. monocytogenes* and *S. aureus* were strongly inhibited at the highest concentrations of chlorogenic acid (48, 35 and 46% respectively).

Regarding gallic acid, the effect on bacterial growth was highly significant on *S. aureus* and *E. sakazakii*, showing a similar inhibition pattern at the highest concentration (92 and 93% respectively). The development of *E. coli* was significantly affected at 0.3–1.25 mg/mL of gallic acid. However, *E. coli* O157:H7 did not show significant (*p* ≤ 0.05) inhibition after exposition to gallic acid. The growth of *L. monocytogenes* was slightly compromised at the highest doses.

The inhibitory effect of ferulic acid (Table 2) showed a considerable interest on *E. sakazakii* and *E. faecalis*. A concentration of 1.25 mg/mL led to a decrease in growth of 90 and 92%, respectively. However, ferulic acid showed a moderate effect on most of the pathogen’s bacteria studied such as *E. coli*, *L. monocytogenes* and *E. coli* O157:H7, reducing their growth around 50%. However, the highest concentration of ferulic acid impaired slightly and moderately the growth of *L. casei rhamnosus* and *L. gasseri,* respectively (Table 4).

Surprisingly, taxifolin showed the strongest anti-listeria effect at concentrations of 1.25 mg/mL with inhibition values of 97%. Difference in the inhibitory effect from 0.6 to 1.25 mg/mL was remarkable. The effect on *E. coli* was also considerable at the highest concentrations and dose dependent. Taxifolin similarly affected the growth of *E. sakazaii* and *E. faecalis* showing a close pattern of growth with inhibition percentages close to 30%. It should be noted that *L. casei rhamnosus* and *L. gasseri* were significantly compromised at concentrations of 1.25 mg/mL for the first one, and 0.6 and 1.25 mg/mL of taxifolin for *L. gasseri*. At the maximum concentration tested, *L. casei rhamnosus* and *L. gasseri*, showed inhibition percentages of 87 and 97%, respectively, (Table 4).

**Figure 2:**
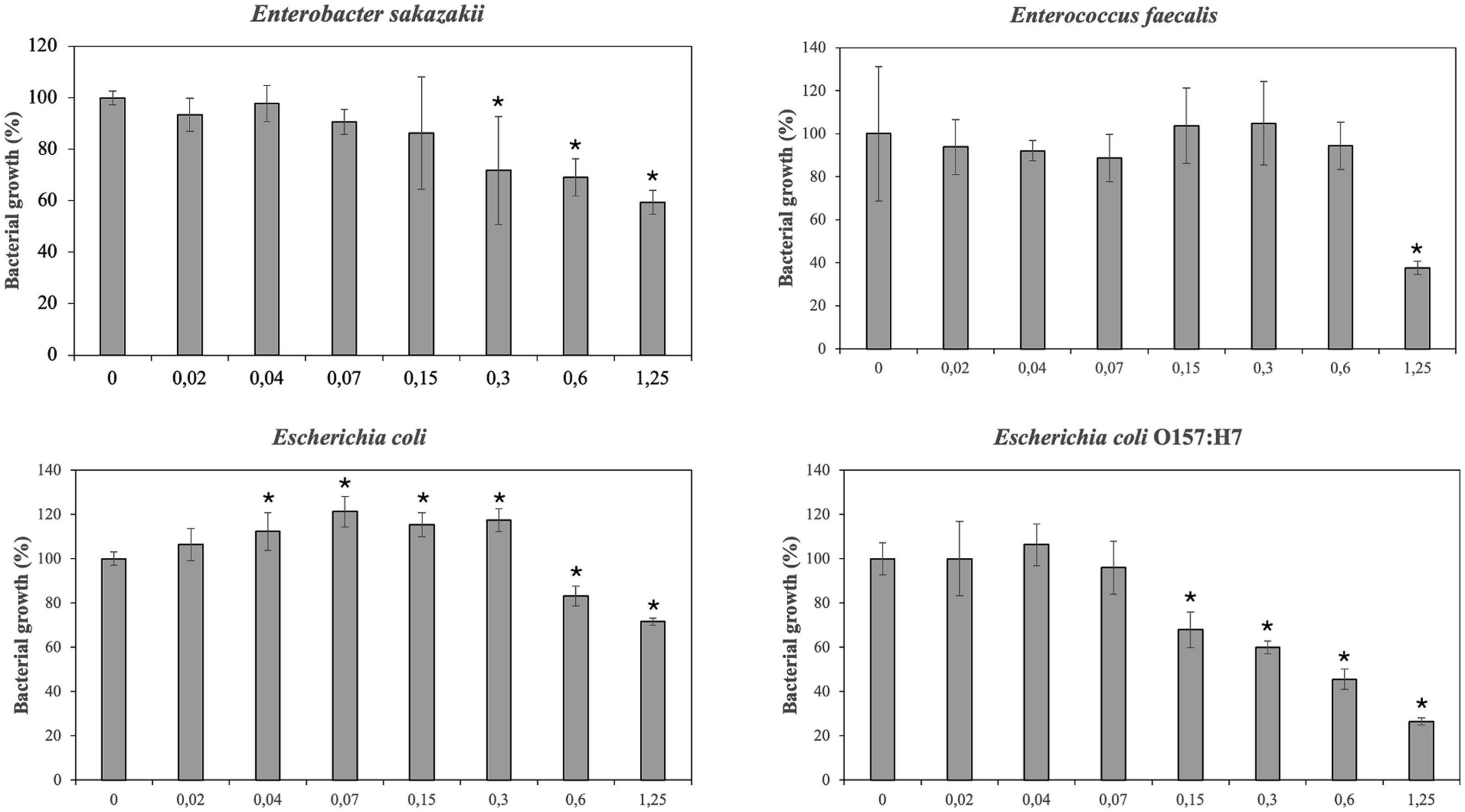
Effect of pine bark extract (PBE) on growth/inhibition on *Enterobacter sakazakii, Enterococcus faecalis, Escherichia coli* and *Escherichia coli* O157:H7.

**Figure 3:**
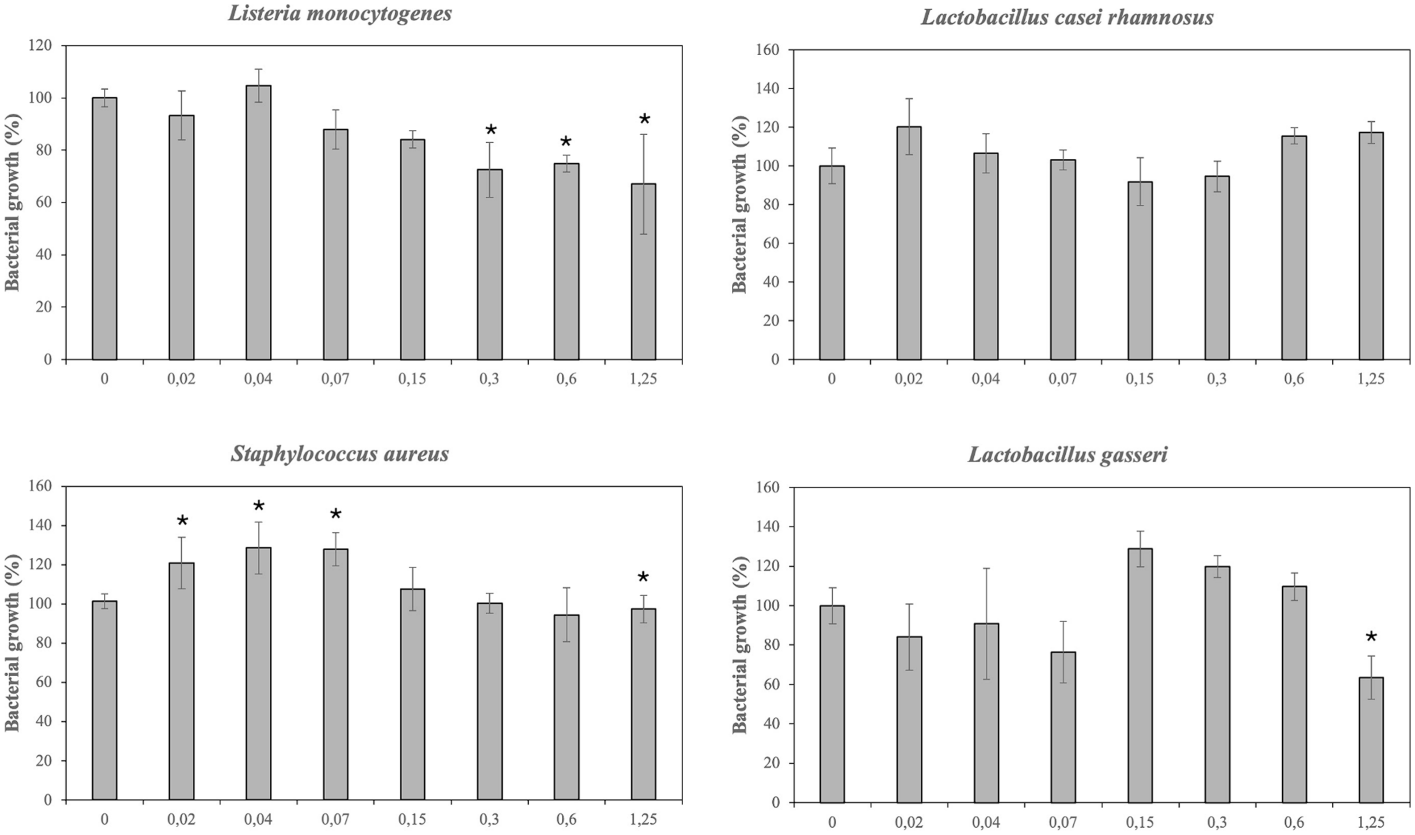
Effect of pine bark extract (PBE) on growth/inhibition on *Listeria monocytogenes, Lactobacillus casei rhamnosus, Staphylococcus aureus* and *Lactobacillus gasseri*.

Antimicrobial effect of PBE has been showed in Figures 2, 3. The inhibitory effect PBE was highly significant on the growth of *E. coli* O157:H7. *L. monocytogenes* was inhibited at maximum concentrations maybe due taxifolin contained in PBE. Furthermore, the growth of *E. faecalis* was reduced 62%, at concentration of 1.25 mg/mL of PBE. The response of *E. coli* after being incubated with PBE was contradictory since some concentrations (0.02–0.3 mg/mL) caused bacterial growth and higher concentrations did the opposite. For beneficial microorganisms, it should be noted that *L. casei rhamnosus* was not affected by the highest concentration of PBE, even showed slightly growth increase. However, the growth of *L. gasseri,* despite growing (0.15–0.6 mg/mL) was compromised at concentration of 1.25 mg/mL of PBE.

Main results of the present work have confirmed that high concentrations of phenolic compounds and PBE have affected the growth of tested bacteria. The inhibitory effect has varied depending on the bacteria species, the phenolic compound, and concentrations. The antimicrobial effect of phenolic compounds can be attributed to their effect on bacterial growth, metabolism and alteration in the functioning of cell membranes (37). Antimicrobial activity of studied compounds may vary depending on the bacterial wall. Gram-negative bacteria appeared to be more resistant to antimicrobial agents due to the hydrophilicity of the surface and associated enzymes capable of breaking down foreign molecules. Besides the presence of negatively charged lipopolysaccharide could protect bacteria against some phenolic compounds (19). In general, data showed that Gram-negative *E. sakazakii* and Gram-positive *E. faecalis* have been very affected by most of the phenolic compounds, specifically by caffeic acid and ferulic acid. On the other hand, Gram-positive lactic acid bacteria were lesser sensitive to PBE and phenolic compounds. The antibacterial activity of caffeic acid has also been observed by different authors (20, 34, 38). In fact, caffeic acid has proven to be one of the phenolic compounds with the greatest antibacterial effect in line to other studies (20). Similar results have been described by López-Nicolás et al. when investigated the effect of caffeic acid on the growth of *E. sakazakii,* showing growth percentages lesser than 50%. As other authors has described, the antimicrobial effect of caffeic acid could be due to a disruption of cell membrane integrity, the bond of phenolics to cell enzyme and alteration of permeability of cell membrane, leading to leakage of cellular constituents (39).

As well as high concentrations of ferulic acid have demonstrated a great effect on bacterial growth. The minimal inhibitory concentration (MIC) can be interpreted as the lowest concentration (μg/mL) that inhibits visible growth of microorganism (40). Shi et al. observed that MIC for ferulic acid against *C. sakazakii* (formerly named *E. sakazakii*) ranged from 2.5 to 5.0 mg/mL. This work showed that the addition of ferulic acid immediately inhibited the bacterial proliferation, causing cell membrane dysfunction and changes in cellular morphology (41). However, ferulic acid showed a low inhibitory effect but not despicable on most of the pathogen’s bacteria studied such as *L. monocytogenes* and *E. coli* O157:H7 in the same manner that other researchers have observed (31). Borges et al. indicated that MIC of ferulic acid for *E. coli* of 100 μg/mL and for *S. aureus* and *L. monocytogenes* (MIC of 1,100 and 1,250 μg/mL, respectively) (40).

Gallic acid has demonstrated to be a potent antimicrobial against *E. sakazakii* and *S. aureus*. Similar to our results, *S. aureus* was very susceptible to high concentrations of gallic acid (0.5 g/L) (31). Furthermore, other researches have also confirmed the antimicrobial effect of gallic acid against Gram-positive bacteria such as *S. aureus* (32, 42). Taguri et al. described the susceptibility of *S. aureus* to hydroxybenzoic acids such as gallic acid (43). In addition gallic acid showed a modest anti-listeria effect similarly to others (32, 44). Chaalal et al. observed the inhibitory effect of gallic against *L. monocytogenes*. In the study performed by Borges et al. the MIC for gallic acid corresponded to 1750 μg/mL for *S. aureus*, 1,500 μg/mL for *E. coli* and 2000 μg/mL for *L. monocytogenes*. Finally, it is worth highlighting that *L. gasseri* was not affected by any tested concentration of gallic acid in line to other works (20, 31).

Taxifolin has demonstrated a notable antibacterial effect. The present data have revealed that high concentrations of taxifolin could reduce the growth of pathogens, such as *E. coli* and *L.monocytogenes*. Jeong et al. reported that MIC of *E. faecalis* for taxifolin was of 0.128–0.512 mg/mL; these values are similar to those obtained in our assay, with bacterial inhibition at 0.15–1.25 mg/mL for *E. faecalis*. The anti-listeria effect of taxifolin was also observed by other authors although to a lesser extent (31). In this regard, Shanti et al. reported that flavonoids like taxifolin had an inhibitory growth effect on *L. casei rhamnosus* at concentration of 0.25 mg/mL. López-Nicolás et al. demonstrated that taxifolin (0.5 g/L) significantly reduced the growth of *L. casei rhamnosus* but not significantly in the in the case of *L. gasseri*. Possibly the antimicrobial effect of taxifolin has been stronger in the case of Gram-positive bacteria (*L. monocytogenes* and *Lactobacillus* strains) than Gram-negative due to its ability to reach the site of action (inducing antibacterial effects through disruption of bacterial cell membranes, generation of reactive oxygen species and interaction with DNA and proteins) (45).

Results have demonstrated that PBE can be useful to reduce the growth of *E. faecalis*, *E. sakazakii*, *E. coli* and *E. coli* O157:H7 probably because pine bark extracts contain a large number of phenolic compounds such as catechins, epicatechins, taxifolin and phenolic acids. In this regard, Ahn et al. reported similar results of inhibition of *E. coli* O157:H7 and *L. monocytogenes* at high concentration of PBE (46). Other researches have observed that PBE (0.5 g/L) had an antimicrobial effect on *E. faecalis* and *E. coli* O157:H7 with growth percentages of 85 and 45% compared to control (31). *Enterobacteriaceae*, including *E. coli*, *E. coli* O157:H7 and *E. sakazakii* cause functional alterations on intestinal mucosa of IBD’s patients, so these results may be relevant. “Pathobionts” can be defined as “a symbiont that is capable to promote pathology only when specific genetic or environmental conditions are altered in the host.” Segmented filamentous bacteria, *Escherichia coli* and *Enterococcus faecalis* could meet the criteria of “pathobionts,” but their pathogenicity seems to depend on the genetic susceptibility of the host and the microbial context. For instance, *E. faecalis* can act as colitogenic and/or protective bacteria depending on the composition of gut microbiota. As well as indicating that “pathobionts” could exert beneficial effect on the host (47). Results have proved that *E. coli* was especially sensitive to the antimicrobial effect of various phenolic compounds, such as taxifolin and PBE. It has been described that susceptibility of *E. coli* to phenolic acids depended on the strain (48). In the present work the growth of non/pathogenic strain of *E. coli* was only inhibited by taxifolin, ferulic acid and gallic acid. Meanwhile the growth of pathogenic *E. coli* O157:H7 was strongly limited by PBE and by caffeic acid, ferulic acid and chlorogenic acid, although to a minor extent.

As it has been described so far, antimicrobial activities of PBE and phenolic compounds have been amply demonstrated by different authors (13, 20, 31, 46, 48, 49). A possible mechanism to explain the antimicrobial action of phenolic acids against pathogens could be summarizes in: (i) destabilization of cytoplasmatic membrane, (ii) the occurrence of local rupture or pore formation in cell membranes, (iii) enzyme inhibition by oxidized products and (iv) by reactions with sulfhydryl groups or formation of reactive quinones that can react with amino acids and proteins (40, 50). General results have demonstrated that phenolic compounds and PBE have an inhibitory effect on pathogenic and commensal intestinal bacteria, protecting probiotic bacteria in some cases. These findings are relevant due to a reduction in microbial diversity in active IBD. In fact, gut dysbiosis is a key factor for the development of IBD with reductions in *Firmicutes* and *Bacteroidetes* and increases of *Proteobacteria* (3). In other words, microbial balance is known to be altered in gastrointestinal dysfunctions such as irritable bowel syndrome and inflammatory bowel disease (51). The growth of probiotic bacteria was poorly affected by the majority of phenolic tested and PBE in the present work. This is quite encouraging as probiotics such *L. casei rhamnosus* and *L. gasseri* benefit the host by improving the intestinal microbial balance and intestinal environment. Increases in probiotics lead to a decrease in the formation of ammonia, skatoles and harmful amine procarcinogens in the large intestine, and reduce acid production that raises fecal pH (20). Moreover, probiotic colonization in the intestine should continue in presence of polyphenol to improve the intestinal microbial balance and inhibit pathogen growth. Thus, restoring gut microbiota imbalance may be a useful strategy to improve microbial diversity in IBD patients.

In view of these results, it is proved that pine bark extract and some of its components have a selective antimicrobial activity on intestinal bacteria, inhibiting pathogen growth and regulating probiotics. The antibacterial activity of PBE could be partly attributed to its constituents. A positive additive and/or synergistic effect could exist between different phenolic compounds present in the PBE. Even though this is an *in vitro* study, the beneficial effect of the phenolic compounds tested can be intuited.

Some limitations of the study can point out, such as conditions not completely physiological since this is an *in vitro* approach. Thus, multiple interactions between diet, digestion, gut bacteria, polyphenols, and products derived from their metabolism have not been considered. However, present results may shed light on the potential of PBE and phenolic compounds modulating microbiota in *in vivo* studies.

## 4 Conclusion

Main evidence derived from the present study indicates that most of the phenolic compounds assessed and PBE have a positive influence on the growth of different bacterial strains that could be found in the gut microflora, what could improve microbial balance. *E. sakazakii* and *E. faecalis* were strongly affected by caffeic and ferulic acid. Gallic acid and taxifolin showed a remarkable effect against *S. aureus* and *L. monocytogenes*, respectively. The inhibitory effect of PBE was highly significant on the growth of *E. coli* O157:H7. As well as caffeic and chlorogenic acids showed a positive effect on probiotic bacteria. PBE could be a good option as ingredient in foods (fruit juices, yogurt, etc.) aimed to modulate the intestinal microbiota of IBD patients. In order to reduce symptoms of IBD, more research, including animal and human studies, are necessary to investigate in depth the antimicrobial and anti-inflammatory properties of PBE added in certain foods.

## Data availability statement

The raw data supporting the conclusions of this article will be made available by the authors, without undue reservation.

## Author contributions

TS-M: Data curation, Formal analysis, Investigation, Methodology, Writing – original draft. RL-N: Conceptualization, Supervision, Writing – review & editing. PP-E: Methodology, Writing – review & editing. CG-B: Data curation, Formal analysis, Writing – review & editing. CF-S: Conceptualization, Project administration, Writing – review & editing.
